# The Werther Effect, the Papageno Effect or No Effect? A Literature Review

**DOI:** 10.3390/ijerph18052396

**Published:** 2021-03-01

**Authors:** Jan Domaradzki

**Affiliations:** Department of Social Sciences and Humanities, Karol Marcinkowski University of Medical Sciences, ul. Rokietnicka 7, 60-806 Poznań, Poland; jandomar@ump.edu.pl; Tel.: +48-618-452-770

**Keywords:** imitation effect, media, media images, media reporting, papageno effect, suicide, werther effect

## Abstract

This paper examines the association between media reporting on suicides and the subsequent suicides. Scientific papers from two online bibliographic sources Medline (PubMed) and PsycINFO were searched. The sample included 108 research papers examining the impact of different types of media stories on suicides. The review revealed that although the media can be a double-edged sword and serve both as a risk and a protective factor, the vast majority of research suggests that the relationship between the media reporting and the actual suicide rates is causal and real. Moreover, both the quantity and the quality of media reporting may trigger additional suicides in society. Simultaneously, research suggests that especially non-fictional presentations of celebrities’ suicides in newspapers and on television news have the biggest influence on the subsequent suicides. Additionally, a strong modelling effect of media reporting on suicide is based on nationality, age, and gender. However, research shows that because a negative reporting style can be modifiable and improved, the media can also have an educative or preventive effect and can reduce the risk of contagion. Consequently, it is important to monitor the implementation of media recommendations for the reporting of suicide, and continuous education of reporters is needed.

## 1. Introduction

Close to 800,000 people die by suicide annually, and for each suicide, there are approximately more than 20 suicide attempts [1]. Consequently, suicide has become an urgent public health problem worldwide and reduction of the suicide mortality rate has been prioritized by the World Health Organization (WHO). Simultaneously, while suicides are influenced by many factors ranging from mental health problems to social factors, one of the research areas on the risk factors is the possible influence of media stories on subsequent suicidal behavior [2]. Interestingly, while in medical literature the idea that media reporting on suicides may lead to suicide contagion dates back to the 19th century [3], the debate itself began in the late 18th century, when Goethe’s novel *The Sorrows of Young Werther* (1774) was followed by numerous suicides across Europe [4]. Nevertheless, although in his classic work *Suicide* (1897) Émile Durkheim argued that some individuals may be prone to suggestion, he did not believe that imitation occurs often enough to affect the suicide rates in society [5]. Consequently, the French sociologist believed that prohibiting newspapers from publishing suicide stories is useless. Thus, until the 1960s, social debate on the copycat effect was based on impressions and it is only in the last 50 years that the effect of media stories on imitative suicides has been recognized as a public health issue and has become a topic of scientific research.

The first evidence on the imitation effect following broad publicization of suicide stories in the media was presented in 1974 by the American sociologist David Phillips, who noted that after stories about suicides had been published in the *New York Times*, their number rose significantly [2]. To indicate the negative influence of media portrayal of suicides, Phillips coined the term the ‘Werther effect’. However, it has been also suggested that under certain conditions, exposure to accounts of suicidal behavior in the media is associated with a lower risk of suicide attempts and can have a more positive, i.e., educative or preventive, effect, especially when the media present constructive coping strategies with suicidal ideations or emphasize other solutions to adverse life circumstances. Indeed, quite recently, to indicate a positive preventive effect of media reporting on suicidal behaviors, Niederkrotenthaler and colleagues [6] introduced the expression the ‘Papageno effect’. The term itself is a tribute to the character from Mozart’s opera *The Magic Flute* (1791), who overcame his suicidal crisis after a talk with three boys. Thus, although the evidence of the impact of media coverage on suicide continues to mount, modern research has been marked by conflicting results [7,8,9,10,11,12,13,14,15,16].

It is of special importance in the context of the increasing prevalence of nonsuicidal self-injurious behavior (NSSI), which is particularly frequent among adolescents and young adults, who are also more prone to imitation. Additionally, even though some perceive NSSI behaviors as an adaptation strategy to a crisis situation or emotion dysregulation [17], it has been well documented that when NSSI behaviors become chronic, they may evolve toward attempted suicides. Moreover, as it has been proven that the relationship between nonsuicidal self-injurious behaviors, suicide attempts, and actual suicides is causal and real [18,19,20], it has also been shown that media portrayals of NSSI may lead to increases in such behaviors [21,22].

Most frequently, the impact of the media on subsequent suicide has been explained either by the social learning theory or by the identification theory [23,24]. The former emphasizes the fact that because most human behaviors are learned observationally through modelling, some vulnerable individuals (for example, patients with depressive disorder, persons with previous suicidal attempts, or those experiencing a psycho-social crisis) may learn from the media that personal problems may be solved by suicide and may adopt suicidal behavior. The latter stresses that because individuals tend to identify with persons who are similar to themselves, persons who face similar emotional states or experience problems or crisis similar to those as suicidal victims presented in the media, may develop a sort of attachment that encourages them to imitate suicidal behavior. At the same time, while some stress the importance of the “horizontal identification”, which indicates that individuals identify more strongly with persons who share their demographic characteristics, such as gender and age [13,14,23,25,26], others refer to “vertical identification” and argue that individuals mimic the behaviors of persons who are famous and popular, admired, or perceived as socially superior, i.e., celebrities [13,26,27,28]. All in all, it is argued that as individuals learn through the media that suicide is an appropriate solution to personal life’s problems, imitation plays an important role in modelling suicides.

Thus, this paper aims to provide an overview of the research referring to the association between media reporting on suicides and the subsequent number of suicidal behaviors in the general population. While previous reviews often focused on a particular type of media (newspaper and television news, television dramas, soap operas, cinema), on the type of suicide (fictional vs. real deaths) or suicide method (i.e., jumping, shooting, railway or subway suicide or paracetamol overdose), on the media effect (the negative vs. the protective effect), or on the social group affected (i.e., patients with depressive disorders, persons with previous suicidal attempts or various demographic groups), this review explores the influence of suicide reporting in various types of media and considers both fictional and real deaths, including celebrity and noncelebrity suicides. It also evaluates the research on both the negative and the protective effect and examines who is more prone to the modelling effect. Thus, it intends to investigate what type of media and what type of suicide reported have the largest influence on imitation suicides.

## 2. Methods

### 2.1. Search Strategy

**Figure 1:**
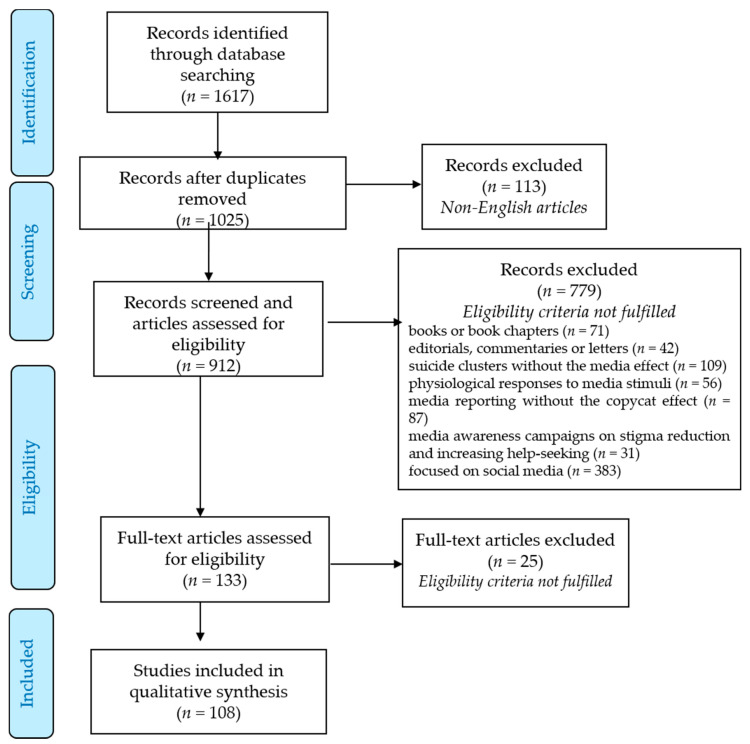
PRISMA 2009 flow diagram for the review and selection process of the studies included.

The review was conducted in accord with the principles of the PRISMA statement [29], and the results of the identification, screening, eligibility assessment, and final inclusion are presented in the PRISMA 2009 flow diagram with some minor modifications (Figure 1). The literature search was conducted in two online bibliographic sources: Medline (PubMed) and PsycINFO in October 2020. For the search criteria, a combination of key words was used: ‘suicide’ (‘suicidal behaviors’, ‘suicidality’) and ‘media’ (‘newspaper’, ‘television’, ‘film’, ‘movie’) either in the title, abstract, or keywords of the publication.

### 2.2. Inclusion and Exclusion Criteria

Articles were included if they reported empirical studies on the impact of media coverage on suicide, were written in English, and were published in peer-reviewed journals. Research on suicide clusters without the media effect, those on physiological and attitudinal responses to suicide films and other media stimuli, and descriptive studies that provided no data on the copycat effect were excluded. Research that focused on the impact of media awareness campaigns on stigma reduction and help-seeking was also rejected. Finally, as this review focused on the traditional media, studies on the impact of the Internet and social media were also excluded.

### 2.3. Study Selection

The initial search identified 1617 publications, which were selected based on their titles and abstracts. After removing duplicates, 1025 records were left for screening. The first stage of the screening excluded all non-English articles (*n* = 113). Next, all other articles were screened on the grounds of their titles and abstracts in order to assess the relevance of the subject and eligibility. Thus, of the remaining 912 publications, 779 did not meet the eligibility criteria: 71 were books or book chapters and 42 were editorials, commentaries, communications, or letters. The additional 109 articles focused on suicide clusters without the media effect, 56 on physiological and attitudinal responses to suicide films or other media stimuli, 87 described media reporting on suicide without the copycat effect, 31 reflected on the impact of media awareness campaigns on stigma reduction and increasing help-seeking, and 383 focused on social media. Thus, of all the studies that met the inclusion criteria, 108 were reviewed and analyzed: 98 research articles, 7 systematic reviews, and 3 meta-analyses.

Such a broad scope of the review has resulted from the research questions posed in the study. As it intended to determine which type of the media and what type of suicide (method) is especially prone to influence the subsequent suicides and whether the media serve as a risk or as a protective factor, research employing a variety of methods was included into the review. However, because of the heterogeneity of the search, the results and the variety of methods used in the studies limited the possibility of a quantitative synthesis of the articles, and both the qualitative and the quantitative findings were integrated and an overall qualitative synthesis was performed and key findings presented.

To assess the papers, the study findings were read and analyzed according to the review questions regarding the role of the media in suicide contagion, the type of the media, and the type of the suicide method reported in provoking subsequent suicides. The qualified articles were assessed using a thematic analysis [30]. To identify the main themes, the study questions were used. The results were selected by employing a deductive approach and classified as thematic categories, representing the relationship between media stories and suicides. Three main themes were identified: (1) the positive association between media portrayal of suicide and imitation acts, (2) the preventive effect of media reporting on suicidal behaviors, and (3) no copycat effect. Nevertheless, not all the articles dealt with every one of these themes.

## 3. Results

The main characteristics and findings of the studies included in the qualitative synthesis are presented in Table 1. Most of the studies (*n* = 45) came from North America, including 41 from the US [2,27,28,31,32,33,34,35,36,37,38,39,40,41,42,43,44,45,46,47,48,49,50,51,52,53,54,55,56,57,58,59,60,61,62,63,64,65,66,67,68] and 4 from Canada [69,70,71,72]. In total, 25 studies were conducted in Asia, including 6 in Taiwan [73,74,75,76,77,78], 8 in South Korea [79,80,81,82,83,84,85,86], 4 in Hong Kong [87,88,89,90], 3 in Japan [91,92,93], 2 in Israel [94,95], one in India [96], and one study was conducted in Hong Kong, Taiwan, and South Korea [97]. Another 23 studies came from different European countries: 10 from the United Kingdom [98,99,100,101,102,103,104,105,106,107], 7 from Austria [6,26,108,109,110,111,112], 4 from Germany [25,113,114,115], and 2 from France [116,117]. Finally, five studies were conducted in Australia [118,119,120,121,122].

In total, 65 studies focused on the impact of newspapers and television news [2,6,26,27,28,31,32,33,34,35,36,37,39,41,43,45,46,49,51,54,55,56,57,59,60,62,63,65,68,69,70,71,72,74,75,76,77,78,79,81,83,84,85,86,87,88,89,90,91,92,93,95,96,97,100,101,102,108,109,110,111,112,113,118,119]; 21 examined the copycat effect following television dramas, soap operas, and other types of movies [25,38,42,44,47,48,50,52,53,58,61,67,94,98,99,103,104,105,106,107,116]; and 12 analyzed the influence of mixed media [40,64,66,73,80,82,114,115,117,120,121,122].

In total, 20 studies investigated the impact of fictional stories on suicides [25,38,42,44,47,48,50,52,53,58,61,67,98,99,103,104,105,106,107,116]; and 78 examined the effect of non-fictional suicides, including those committed by celebrities [2,6,26,27,28,31,32,33,34,35,36,37,39,40,41,43,45,46,49,51,54,55,56,57,59,60,62,63,64,65,66,68,69,70,71,72,73,74,75,76,77,78,79,80,81,82,83,84,85,86,87,88,89,90,91,92,93,94,95,96,97,100,101,102,108,109,110,111,112,113,114,115,117,118,119,120,121,122].

In total, 69 reported the negative impact of the media on the actual suicide rates [2,25,26,31,32,33,34,35,36,37,38,39,43,44,46,47,48,49,51,56,57,59,60,61,62,63,67,68,70,71,72,73,74,75,76,77,78,79,80,81,82,83,84,85,86,87,88,89,90,91,92,93,96,97,100,101,102,103,104,107,111,113,114,115,116,118,119,121,122], and 16 found no imitative effect [31,40,41,42,45,50,53,54,55,58,64,69,95,105,106,120] while only 4 reported the protective effect, which followed either the cessation of stories during newspaper strikes or the implementation of media guidelines for suicide reporting [108,109,110,112]. Additionally, nine studies found mixed or conflicting results [6,27,28,65,66,94,98,99,117].

### 3.1. The Werther Effect

The first research indicating a positive correlation between media stories about suicides and subsequent deaths was described by Phillips in 1974, who showed that suicide stories published in the *New York Times* influenced the actual number of suicides [2]. In later studies, Phillips reported that imitational suicides may also be hidden as motor vehicle fatalities [34,36,37] or airplane accidents [35] and that their number increased after suicide stories appeared in newspapers and on television. This debate was further advanced when Bollen and Phillips [39] and Phillips and Carstensen [46,51] found a significant increase in suicides among American teenagers during the week following the broadcasting of suicide stories on network television evening news programs between different time periods (1972–1976, 1973–1979, and 1968–1985, respectively) [38,43].

Similarly, the vast majority of research (*n* = 69) included in this review shows a positive association between media coverage of suicidal behaviors and actual suicidality. At the same time, although researchers have examined the modelling effect following the various types of media, including newspaper and television news, television dramas, soap operas, and cinema, reviews by Stack [13,123] suggested that televised stories were 82% less apt to find the imitation effect than the research based on newspapers. On the other hand, while major television networks had a larger influence on imitation suicides than those covered by other types of news media, such a probability was even higher when suicides were reported by multiple media. Moreover, stories on non-fictional suicides, i.e., those committed by real people, and celebrities in particular, were found to be more likely to influence suicidal behavior than fictional suicides depicted in movies, TV dramas, or weekly soap operas (see below) [7,8,9,10,11,12,13,14,15,16]. For example, Stack [123] reported that non-fictional stories were 4.03 times more likely to predict the copycat effect than fictional stories and that stories about celebrity suicides were 14.3 times more likely to provoke the Werther effect. However, some studies reported increased rates in suicides following the exposure to fictional dramatizations of suicides in movies [8,11,25,38,44,47,48,52,61,67,103,104,107,116].

Because the impact of non-fictional suicides is discussed in the next paragraph, let us now present only some general findings on fictional portrayals of suicide in movies. Thus, in the 1980s, Ostroff et al. [44,48] reported an increased number of young people who attempted suicide after the airing of a TV drama *Surviving* showing a teenage couple who committed suicide by overdose. Additionally, all respondents they interviewed reported having seen the program. Schmidtke and Häfner [25] also reported the Werther effect after the broadcast of a German fictional weekly TV series featuring the railway suicide of a 19-year-old male. Moreover, they reported both increased suicide rates and the number of suicides committed by the same method. The same effect was observed when the series was repeated years later.

Biblarz et al. [61] demonstrated that college students exposed to movies about suicide showed significant emotional arousal and changes in the attitude toward suicide. Hawton et al. [107] reported that a TV broadcast *Casualty* depicting a deliberate paracetamol overdose influenced young people’s self-poisoning, which increased by 17% in the week after the airing of the episode. Moreover, 20% of patients declared that the program had influenced their decision to take an overdose and 17% said it had influenced their choice of the drug. All in all, while it is often suggested that non-fictional suicide stories in newspaper or television news are more likely to provoke the copycat effect, fictional suicides depicted in movies may also provoke imitative behaviors. At the same time, it has been shown that there are some conditions that enhance the probability of the imitation effect: the celebrity status of the suicide victim, similar demographic characteristics of the victim and the media audience, and media reports on a new suicide method.

As it has been found that in order to influence the public, suicide stories must be about real and not fictional people, research also suggests that it is famous and socially admired individuals who are particularly strong role models and give some sense of legitimacy to the suicidal act [2,27,28,49,57,92]. For example, Jonas [113] reported that each suicide committed by a prominent individual publicized in major German newspapers was associated with additional suicides during the week after the report. Similarly, both Fu and Yip [97] and Park et al. [85] showed that celebrity suicides constitute an independent risk that may trigger suicidal ideation not only among individuals experiencing depression or negative life events but in anyone. A review conducted by Niederkrotenthaler et al. [26] found that the celebrity status of the reported suicide was the major predictor of a post-report increase in total suicides. Additionally, a more recent meta-analysis revealed an association between media stories of a celebrity suicide and the subsequent suicides, which increased on average by 2.6 suicides per 100.000 people within the month following such reports [15,16].

Thus, especially during the last 20 years, news reporting about celebrity suicides has been at the core of scientific interest in the media impact on the copycat effect [8,10,11,15,16,26,70,71,72,73,74,79,80,81,82,83,84,85,86,88,91,96,97,111,114,117,120,121,122,123]. For example, Ladwig et al. [115] showed that after the railway suicide by a famous German national goalkeeper Robert Enke, the overall percentage of suicides increased by 117.2%. Ueda et al. [93], who investigated the impact of media reporting on 109 celebrity suicides in Japan from 1989 through 2010, found that such reports resulted in an immediate increase in the number of suicides in the general population by 4.7%. Additionally, a recent review by Niederkrotenthaler et al. [16] reported that media stories about celebrity suicides have a strong impact on the total number of suicides in the general population.

However, research suggests that the imitation effect holds mainly for two types of celebrities: entertainers (i.e., music and movie stars) and political figures, while famous villains and the economic elite do not trigger imitative suicides [8,13,15,28,49,70,72,73,75,79,80,85,88,93,97,117,123]. For example, Stack [8] reported that the impact of entertainment or political celebrity suicide reports was 14.3 times more likely to trigger the copycat effect than other stories. Additionally, his other meta-analysis found that such suicides were 55.27 times more likely to produce the imitation effect [13]. A similar observation was reported by Niederkrotenthaler et al. [15]. These general findings were supported by Queinec et al. [117], who investigated the impact of media coverage of six famous celebrity suicides between 1979 and 2006 in France and found the modelling effect after the suicides of the famous singer Kurt Cobain and the politician Pierre Bérégovoy, but not in the other four. The suicide committed by the famous actor Robin Williams was followed by a 10% increase in US suicide rates, especially among males aged 45–64 years, and many individuals used the same method [71,122]. Menon et al. [96] reported that more than 5% of the total suicides in India were linked to the widely reported death of the celebrity actor Sushant Singh Rajput.

Nevertheless, some research has shown that not all entertainment celebrity suicides reported by the media increased the actual suicides rates. For example, although in Australia the death of the famous singer Kurt Cobain received wide media attention, owing to its cautious reporting style, the Werther effect did not occur [64,120]. Additionally, Fu and Chan [80] found that apart from intensive media coverage, only 3 of 11 Korean celebrity suicides had a significant impact on the overall suicide rates.

Yet another study suggests that noncelebrity suicides, too, may trigger the imitation effect as people who commit suicide can also identify themselves with ordinary people, i.e., persons of a similar background or social status [13,14,23,25,26,49,56,90]. For example, Kunrath et al. [114] reported a 44% daily increase in railway suicides after media coverage of a non-prominent railway death. This supports Stack’s [56] and Gundlach and Stack’s [60] observation that if a story about noncelebrity suicide receives enough publicity, it can also provoke imitation. Thus, arguably, it is not the unique quality of the celebrities’ status *per se* that leads to imitation but rather high publicization of such a suicide that assures the celebrity status of a suicidal person.

At the same time, most studies report that especially persons who experience emotional or mental problems and share the suicide victim’s demographic characteristics or are in similar life situations are more likely to imitate suicidal behavior. It is argued that particularly vulnerable individuals, i.e., those with pre-existing psychopathology or in a psycho-social crisis (suffering from depressive disorder, with previous suicidal ideations or previous suicidal attempts), are at an increased risk of the modelling effect [8,9,13,14,16,25,26,59,68,79,123]. For example, Fu and Yip [97] and Park et al. [85] reported that individuals experiencing depression or negative life events are more likely to imitate celebrity suicides publicized in the media.

Furthermore, the copycat effect was more notable in the groups whose age, gender, and nationality were similar to those of the victim. For example, as domestic celebrities were found to have a greater potential to provoke the imitation effect than foreign celebrities [8,15,28,123], both Stack [92] and Choi and Oh [84] reported that while Japanese and Korean victims triggered subsequent suicides in the general population, non-Japanese and non-Korean suicides did not.

Furthermore, Fu and Yip [97] reported an elevated risk in terms of the same age (49%), gender (40%), and method (63%). However, although some argue that all age groups are prone to the copycat effect [79,85], both Stack [13] and Chen et al. [75] reported a significantly higher risk among the middle-aged, while Stack [8,57,123] and Sinyor et al. [72] found evidence that media stories about elderly suicides were followed by individuals aged 65 and over. They stressed that it may have resulted from the suicidogenic life circumstances of the elderly, i.e., a high degree of physical illness, deaths of spouses and friends, social isolation, and low income. Similar observations were made in Taiwan [75].

Nevertheless, it is younger people in particular, including adolescents, who were found to be the most vulnerable to imitative influences [10,12,13,25,26,44,46,47,48,51,54,55,66,68,71,72,75,79,80,86,87,88,96,97,120]. For example, Hawton et al. [107] found out that TV broadcasts picturing a deliberate paracetamol overdose increased young people’s self-poisoning by 17%. Additionally, Gould et al. [12,68] showed that the suicide risk among 15–19-year-olds exposed to suicide reports was 2 to 4 times higher than among other age groups. According to Stack [13], research results based on young persons were 54% more likely to find the copycat effect.

Although some researchers have found no gender-specific association [88], and there is a body of evidence showing that the imitative effect is more prominent among persons of the same gender as the suicide victim [75,79,88,97]. At the same time, while some studies report that males are more susceptible [71,73,88,97], other suggest a much greater imitation effect among females [2,16,26,73,75,79,80,88,96,97]. For example, as early as in the 1970s, Barraclough, Shepherd, and Jennings [100] reported an increased risk of imitation in men under 45. A similar observation was made in Australia [118]. Additionally, Fink et al. [71] reported a 10% increase in US suicides, especially among men aged 45–64 years, in the months following the suicide of Robin Williams [122]. On the other hand, Phillips and Carstensen [46,51] found a significant increase in suicides among American female teenagers following the airing of suicide stories on television evening news, and, according to Stack [13], studies were 4.89 times more likely to find the copycat effect among women. A recent study in India reports that young females are more susceptible to the imitation effect [96].

Finally, research has shown that, as the media influences the increase in the total number of suicides, they also boost the probability of using the same method as the victim. Such a probability is especially high when the suicide method was used by a celebrity, reported in detail and as novel or painless [15,75,80,81,97,123]. Some researchers even suggest that after media reports on novel suicide methods, the number of those employing conventional methods dropped [76,78]. Indeed, a recent review of 31 studies published between 1974 and 2019 found that media descriptions increase the cognitive availability of a suicide method used by a celebrity, and this, in turn, results in a 30% increase in deaths by the same method [16]. Thus, while Fu and Yip [97] reported an elevation in the risk of suicide in terms of the same method by 63%, many researchers have found that media stories were associated with an increased number of suicides employing methods, such as burning [101,102], charcoal burning [75,76,77,78,87,89,90], hanging [16,70,73,79,96], shooting [111,120], plastic bag asphyxia [116], jumping [79,88,94], railway suicide [25,114,115], subway suicide [108,109,110], suffocation [71], and paracetamol overdose [44,48,103,104,105,107]. For example, Ostroff et al. [44] and Ostroff and Boyd [48] observed an increased number of young people admitted to a Connecticut hospital who attempted suicide by overdose, the method used by a teenage couple in a TV drama *Surviving*. An interview-based study from Taiwan reported that 87% of individuals who survived a suicide attempt by charcoal burning admitted that their choice of the burning method was influenced by the media [75,76,87]. A similar observation was made in Hong Kong [89,91] and in South Korea, where the suicide rates by hanging and jumping increased by 31.80% and 60.71%, respectively, after the media reported about the actress Jin-Sil Choi and former President Moo-Hyun Roh of the Republic of Korea who used those two suicide methods [79]. Finally, both Fink et al. [71] and Pirkis et al. [122] found that the suicide rates by hanging increased significantly after the death of Robin Williams.

At the same time, while some researchers have shown that imitation exerts rather a short-term effect [2,25,39,46,74,83,98,99,111,113], others suggest that it may also trigger a long-term effect [15,70,71,77,79,80,91,97,114,115]. For example, while Bollen and Phillips [39] found two significant post-report peaks in suicides, one immediately after the media reports and another after 10 days, other researchers have established that the media effect can persist for 2 [44,48,75,93], 4 [2,73,77,81,97,115], 6 [79], 8 [94,114], or even 9 weeks [80].

### 3.2. The Papageno Effect

Even though earlier researchers were skeptical of the possible preventive effect of media reporting on suicide [31], it was Jerome Motto who first demonstrated that suicide rates may be reduced either by a newspaper blackout, lowering of the quantity of reporting, or by changing the quality of the media reporting style, i.e., by elimination of the emotional and sensational details of suicidal behaviors. He found that the 286-day period of a newspaper blackout in Detroit resulted in a reduction of suicide rates among young females [32]. A similar observation was made by Blumenthal and Bergner [33], who reported a decrease in suicides following the cessation of news stories that occurred during newspaper strikes. Finally, early studies by Holding [98,99] showed no increase in suicide attempts following an 11-part series on a suicidal individual who was helped by the *Samaritans*, the prevention service in Edinburgh. Moreover, the series increased the audience’s knowledge on the suicide prevention center and the number of its clients. Consequently, it has been argued that when the media present constructive coping strategies with suicidal ideation or emphasize other solutions to adverse life circumstances they can have a preventive effect [6,14,16,108,109,110,112].

Simultaneously, while some researchers suggest that the media support suicide preventive efforts by educating the public about the treatment of mental illness [124], others stress that it is rather negative media coverage of suicides, i.e., the ‘non-attractiveness’ of the suicide victim or the circumstances of the suicidal act that decrease the probability of the imitative effect [36,55]. Indeed, researchers have found that when the media describe suicide via unfavorable characteristics, i.e., as a wrong, horrific, painful, or disfiguring act, or present suicidal individuals as deviants, it may weaken the probability of imitation [9,13,26,68,73]. For example, negative media coverage of the Jonestown mass suicide in 1978 [40] and Kurt Cobain’s death in 1994 [64,120] was linked to the absence of the copycat effect. Additionally, Romer, Jamieson, and Jamieson [66] observed that although newspaper reports and television news were associated with an aggregate increase in suicide deaths, especially among younger individuals, in persons aged 25–44 they had a protective effect. Finally, Niederkrotenthaler et al. [6] found that reports on individuals who contemplated suicide but later coped with the adverse circumstances in a constructive way were associated with a short-term decrease in suicide rates.

However, a systematic review 56 studies on the role of the media in suicide prevention by Sisask and Värnik did not confirm the protective effect [14]. Additionally, Sinyor et al. [72] reported that the most putative protective factors, such as unfavorable characteristics of the suicidal act or spreading messages that suicidal thoughts can be overcome, have low base rates in the media and are not associated with a lower number of suicides.

### 3.3. The ‘No Effect’

Although the majority of researchers have found the Werther effect following media reporting, a considerable number of 26 studies have noted either conflicting results [6,27,28,65,66,94,98,99,117] or no copycat effect at all [31,40,41,42,45,50,53,54,55,58,64,69,95,105,106,120]. Likewise, Stack reported that of 42 research findings, less than half were significant and that highly publicized stories increased the national suicide rate only by 2.51% [8]. Additionally, his other meta-analysis showed that out of 419 findings, only 35.8% gave evidence on the imitative effects following media reports on suicide [13].

Earlier studies, in particular, failed to demonstrate the convincing effect of media reports on real suicides. For example, after replicating Phillips’ [38] research, Kessler and Stipp [42] were unable to discover any evidence on the impact of soap opera stories on the actual amount of suicides. In the same way, Wasserman [27], who re-examined Phillips’s [2] data, found no significant linkage between the *New York Times*’ front page stories on suicide and the subsequent number of suicides, except for celebrity suicides. Additionally, Hittner [65] obtained little or no evidence to support the Werther effect reported by Phillips and Carstensen [46,51]. Finally, Stack [40] did not observe any relationship between media reporting of the mass suicide in Jonestown, Guyana, and the national suicide rates. Moreover, some authors suggest that the increased suicide rates are better explained by an economic rather than imitation paradigm [28,41,63].

At the same time, while most of those suicides were committed before 1990, some more recent investigations have also found no such association [80,117]. For example, Simkin et al. [106] did not find evidence of the impact of a TV drama *Casualty* showing a teenage girl’s overdose with paracetamol either on the number of patients’ decisions to take an overdose or their choice of drug. Bakst et al. [95] also did not observe any impact following media reporting about 13 deaths on the subsequent number of suicides in Israel.

## 4. Discussion

This review confirms that there is a growing amount of evidence suggesting a positive association between media coverage of suicidal behaviors and the actual suicidality [7,8,9,10,11,12,13,14,15,16]. Although a considerable number of negative findings has cast some doubt on the existence of a media-induced imitative effect [40,41,42,45,50,53,54,55,58,64,69,95,105,106,120], 69 studies included in this review report that media stories on suicide are contagious and may influence the actual suicide rates. Significantly, while before the 1990s most of the studies were conducted in the US, after 2000, the number of investigations from other countries has increased rapidly. It is important because the quality of media reporting on suicide varies from country to country and reflects the differences in social attitudes and ideas about life, death, and suicide embedded in culture. For example, Fekete et al. [125] found that negative evaluations of suicide are much more common in American, Finnish, and German newspapers, which tend to depict suicides as criminal or psychiatric in nature and describe the more negative consequences of suicidal behaviors. Meanwhile, Hungarian and Japanese press characterizes suicides in a more romantic manner. Thus, while the copycat effect is observed in various countries worldwide, it has been shown to be dependent on the country where the study was conducted [15,16,87].

Although the impact of various kinds of media has been examined, it seems that newspaper stories have the largest influence. Simultaneously, stories presented visually may also influence suicidal ideations and behaviors [121]. However, the copycat effect is even bigger when suicides are reported by multiple media [13,123].

Another important issue is that presentations of real-life suicides and those committed by famous and socially admired people, in particular, are more likely to trigger additional suicides than fictional suicides depicted in television dramas, soap operas, and the cinema [16,72,84,85,86,91,96,122,126]. Nevertheless, because, under certain conditions, non-celebrities may also become role models for imitation, it has been suggested that broad publicization and the quality of media reporting are more important for provoking the copycat effect. Thus, the Werther effect depends more on the duration, amount, and prominence of media coverage [13,73,77,83,97,121].

It has also been observed that a strong modelling effect is based on nationality, age, and gender. Thus, young persons and individuals with previous suicidal ideations or attempts are especially more vulnerable to the imitation effect [8,15,16,26,28,68,79,85,86,123]. Moreover, while some studies have reported a short-term effect, lasting from a couple of days to up to two weeks [2,39,44,46,48,74,75,83,98,99,111,113], the media can also trigger a long-term effect, which can persist for eight to nine weeks [15,77,79,80,91,114,115].

Many researchers emphasize that although suicide is a complex phenomenon, media reports on suicide are selective and oversimplify the causes of suicide. While the factors affecting suicide range from physical and mental health problems, financial problems, broken (family) relationships, failure in school, substance abuse, and peer influence, to stressful life events, the media tend to attribute the act to a single factor. Moreover, they often underreport the relationship between suicide and mental illness, which is the most common factor leading to suicide [6,125]. For example, Gould [9] reported that many Hungarian newspapers characterized suicides in a romantic manner, which resulted in higher rates of suicidal acts in Hungary. Similarly, after analyzing 1377 cinematic suicides appearing in 1158 American movies released between 1900 and 2009, Stack and Bowman [67] found that in contrast to the scientific literature, which attributes suicide mainly to mental health issues, the cinema provides a highly sociological definition of suicide: it is attributed to economic or social strain, rejection in love, relationships falling apart, or shame. Other media also suggest that suicides are caused by societal problems [6].

Moreover, the media reporting style was described as sensational and characterized by dramatic coverage, glorification, or glamorization of the deceased. For example, an analysis of Swiss press shows that 40–45% reports described suicides irresponsibly: 47% used sensational headings (large print, located on the front page, and sensational content), 39.1% presented at least one picture of the victim, the text of 35.8% articles was sensational, and 8.6% were front page articles [127,128]. A similar observation has been made in other countries, where the media tend to report suicides in a romanticized, glorifying, or thrilling manner. Moreover, the media often depicted the victim as an extraordinary or popular personality and suicide itself was framed as an inevitable or honorary act of bravery. The positive image of suicide was further strengthened by rationalization or romanticization of the victim’s motives, for example, by describing it as a result of a terminal or painful illness, financial crisis, job loss, or heartbreak, which in the eyes of the public can legitimatize suicide [68,72,111,124].

Finally, most studies report that media stories on suicide do not reflect the real incidence of suicidal acts as they often focus on the more violent modes of suicide that are presented in a more dramatic fashion [125,129,130,131]. Consequently, there is an overrepresentation of some kinds of suicides in the media. Pirkis et al. [130] find that only 1% of Australian suicides were newsworthy and became public via the media. Thus, they noticed an over-reporting of suicides by drowning (6% vs. 2% of all actual suicides), gunshot (18% vs. 10%), and high-impact methods (12% vs. 7%). Moreover, while suicides committed by the elderly are also over-reported (20% vs. 13%), those committed by the young are under-reported (26% vs. 21%). Fu, Chan, and Yip [132] reported that Chinese newspapers over-publicize suicides committed by persons aged under 25 and underplay those by persons aged 60 or over. More importantly, most studies stress over-reporting of suicides committed by celebrities [127,128,130,132]. Thus, there is also a growing amount of evidence showing that extensive media reporting contributes to the ‘advertising’ of the unusual, novel, or dramatic and highly lethal suicide methods, which are rare in real life, such as burning, plastic bag asphyxia, or suffocation [16].

At the same time, especially since the 1990s, researchers have suggested that, under certain conditions, the media may also play a positive, i.e., educative and preventive role [6,14,16,108,109,110,112]. There is also some evidence that the negative influence of the media may be modifiable and that improving the quality of suicide reporting reduces the risk of contagion. In fact, ever since WHO developed the media recommendations for suicide reporting in 2000, they have been implemented in several countries, including Australia, New Zealand, the United States, Canada, Germany, Austria, Switzerland, the United Kingdom, Japan, Hong Kong, and China [133]. Moreover, while such guidelines have positively influenced media reporting on suicidal behavior [134,135], it has also been proven that modification of the media reporting style may reduce the risk of the Werther effect [6,26,64,132,133,134,135,136,137,138,139,140]. For example, after implementation of the media recommendations in Austria, the suicide rates in the Viennese subway declined by 75% and remained low for more than 5 years [108,109,110]. In Australia, it resulted in a reduction of 81 suicides [112]. Thus, it seems that the role of the media is more complex, as it can be both a risk and a protective factor. However, the media often do not follow these recommendations: suicide stories frequently appear on the front page, include inappropriate imagery, mention suicide in the headline, describe the suicide method and the location, fail to link the death to broader social issues, and do not provide information about the warning signs, risk factors, and prevention resources [122,124,125,126,127,128,129,130,131,132,133,134,135,136,137,138,139,140].

Although this review brings some new insight into the impact of media reporting on the subsequent suicidal behavior, it also has some limitations. First, the investigation was limited to two databases and some studies could not be identified. Second, it only analyzed articles written in English and published in peer-reviewed journals. Thus, in the future, it would be advisable to also include some non-English research and those materials published as books or book chapters. However, owing to the large amount of research included, this limitation should not change the general view and conclusions. Another limitation stems from the fact that the studies selected for the review referred to different kinds of media and/or were conducted on different populations. Thus, it is hard to make quantitative comparisons and draw conclusions.

Finally, as only one coder screened the papers and conducted the analysis, it may have affected the reliability of the coding process. However, despite these limitations, a qualitative analysis was still possible and justified. Yet another limitation stems from the reviewed research itself. Because many studies were designed as ecological research, they failed to provide clear evidence that individuals who engaged in suicidal behavior following an event being reported in the media had actually been exposed to it. Thus, future research, both on the negative and the positive impact of the media, should try to determine whether suicidal individuals actually had access to the stimulus implied. Finally, as much more research has been done on the ‘Werther effect’ than on the ‘Papageno effect’, there is a risk of a reporting bias.

## 5. Conclusions

While the media can be a double-edged sword and serve both as a risk and a protective factor, there is a growing amount of evidence showing that the relationship between media reporting and the actual suicide rates is causal and real. Consequently, the influence of media reporting on suicide is defined as an important risk factor affecting suicidality in the general population. At the same time, researchers suggest that both the quantity and the quality of media reporting may trigger additional suicides. Thus, suicide contagion is more likely to occur after extensive media coverage with a content rich in positive definitions of suicide. Moreover, especially non-fictional presentations of celebrity suicides can strongly influence the subsequent suicidal behavior. However, it should be acknowledged that because the negative reporting style can be modifiable and improved, the media can also have an educative or preventive effect and can reduce the risk of contagion. Consequently, it is important to monitor the implementation of the media recommendations for the reporting of suicide, and continual education of reporters is needed.

## Figures and Tables

**Table 1:** Studies examining the impact of the media on imitative suicides.

Source	Country Studied	Media Studied	Type of Suicide	Main Findings
[2] Phillips 1974	USA	Newspapers	Real suicides	Werther effect
[6] Niederkrotenthaler et. al. 2010	Austria	Newspapers	Real suicides	Mixed results
[25] Schmidtke and Häfner 1988	Germany	Six-episode TV movie	Fictional suicides (railway suicide)	Werther effect
[26] Niederkrotenthaler et al. 2009	Austria	Newspapers	Real suicides	Werther effect
[27] Wasserman 1984	USA	Newspapers	Real suicides	Mixed results (Werther effect for celebrity suicide)
[28] Stack 1992	USA	Newspapers	Real suicides	Mixed results
[31] Motto 1967	USA	Newspaper (cessation)	Real suicides	No effect
[32] Motto 1970	USA	Newspaper (cessation)	Real suicides	Werther effect (age/gender specific)
[33] Blumenthal and Bergner 1973	USA	Newspaper (cessation)	Real suicides	Werther effect (age/gender specific)
[34] Phillips 1977	USA	Newspapers	Motor vehicle fatalities	Werther effect
[35] Phillips 1978	USA	Newspapers	Airplane accident	Werther effect
[36] Phillips 1979	USA	Newspapers	Motor vehicle fatalities	Werther effect (age specific)
[37] Bollen 1981	USA	Newspapers	Motor vehicle fatalities	Werther effect
[38] Phillips 1982	USA	TV soap opera	Fictional suicides (Motor vehicle fatalities)	Werther effect
[39] Bollen and Phillips 1982	USA	TV network news	Real suicides	Werther effect
[40] Stack 1983	USA	Mixed media	Real suicides	No effect
[41] Horton and Stack1984	USA	TV network news	Real suicides	No effect
[42] Kessler and Stipp 1984	USA	TV soap opera (replication of Phillips 1982 [38])	Fictional suicides (Motor vehicle fatalities)	No effect
[43] Phillips 1985	USA	Newspapers	Real suicides	Werther effect
[44] Ostroff et al. 1985	USA	Fictional telemovies	Fictional suicides	Werther effect (age specific)
[45] Baron and Reiss 1985	USA	TV network news	Real suicides	No effect
[46] Phillips and Carstensen 1986	USA	TV network news	Real suicides	Werther effect (age specific)
[47] Gould and Shaffer 1986	USA	TV movies	Fictional suicides	Werther effect (age specific)
[48] Ostroff and Boyd 1987	USA	Fictional telemovies	Fictional suicides	Werther effect (age specific)
[49] Stack 1987	USA	Newspapers	Real (celebrity) suicides	Werther effect (celebrity effect)
[50] Phillips and Paight 1987	USA	TV movies	Fictional suicides	No effect
[51] Phillips and Carstensen 1988	USA	TV network news	Real suicides	Werther effect (age specific)
[52] Gould and Shaffer 1988	USA	TV movies (Replication of Gould and Shaffer 1986 [47])	Fictional suicides	Werther effect (age specific)
[53] Berman 1988	USA	TV movies	Fictional suicides	No effect
[54] Kessler et al. 1988	USA	TV network news	Real suicides	No effect
[55] Kessler et al. 1989	USA	TV network news	Real suicides	No effect
[56] Stack 1990	USA	Newspapers	Real (non-celebrity) suicides	Werther effect
[57] Stack 1990	USA	TV network news	Real suicides	Werther effect (age specific)
[58] Stack 1990	USA	TV movies	Fictional suicides	No effect
[59] Stack 1990	USA	Newspapers	Real suicides	Werther effect
[60] Gundlach and Stack 1999	USA	Newspapers	Real suicides	Werther effect
[61] Biblarz et al 1991	USA	Movie	Fictional suicide	Werther effect
[62] Wasserman 1992	USA	Newspapers	Real suicides	Werther effect
[63] Stack 1993	USA	TV network news	Real suicides	Werther effect
[64] Jobes et al. 1996	USA	Mixed media	Real (celebrity) suicides	No effect
[65] Hittner 2005	USA	TV network news (Replication of Phillips and Carstensen 1986 [46] & 1988 [51])	Real suicides	Mixed results
[66] Romer et al. 2006	USA	Mixed media	Real suicides	Werther effect/Mixed results
[67] Stack and Bowman 2011	USA	Movies	Fictional suicide	Werther effect
[68] Gould et al. 2014	USA	Newspapers	Real suicides	Werther effect (age specific)
[69] Littman 1985	Canada	Newspapers	Real suicides	No effect
[70] Tousignant et al. 2005	Canada	Newspapers	Real suicides	Werther effect
[71] Fink et al. 2018	Canada	Newspapers	Real (celebrity) suicides	Werther effect
[72] Sinyor et al. 2018	Canada	Newspapers	Real suicides	Werther effect
[73] Cheng et al. 2007	Taiwan	Mixed media	Real (celebrity) suicides	Werther effect (gender/method specific: hanging)
[74] Chen et al. 2011	Taiwan	Newspapers	Real suicides	Werther effect
[75] Chen et al. 2012	Taiwan	Newspapers	Real (celebrity) suicides	Werther effect (age/gender/method specific: charcoal burning)
[76] Chen et al. 2013	Taiwan	Newspapers	Real suicides	Werther effect (method specific: charcoal burning)
[77] Yang et al. 2013	Taiwan	Newspapers	Real suicides	Werther effect (method specific: charcoal burning)
[78] Chang et al. 2015	Taiwan	Newspapers	Real suicides	Werther effect (gender/method specific: charcoal burning)
[79] Kim et al. 2013	South Korea	Newspapers	Real (celebrity) suicides	Werther effect (method specific: hanging and jumping)
[80] Fu et al. 2013	South Korea	Mixed media	Real (celebrity) suicides	Werther effect
[81] Lee et al. 2014	South Korea	Newspapers	Real (celebrity) suicides	Werther effect
[82] Ju et al. 2014	South Korea	Mixed media	Real (celebrity) suicides	Werther effect (age/gender specific)
[83] Suh et al. 2015	South Korea	Newspapers	Real (celebrity) suicides	Werther effect
[84] Choi et al. 2016	South Korea	Newspapers	Real (celebrity) suicides	Werther effect
[85] Park et al. 2016	South Korea	Newspapers	Real (celebrity) suicides	Werther effect
[86] Lee 2019	South Korea	TV network news	Real suicides	Werther effect
[87] Chung and Leung 2001	Hong Kong	Newspapers	Real suicide	Werther effect (age/method specific: charcoal burning)
[88] Yip et al. 2006	Hong Kong	Newspapers	Real (celebrity) suicides	Werther effect (age/method specific: jumping)
[89] Yip and Lee 2007	Hong Kong	Newspapers	Real suicides	Werther effect (method specific: charcoal burning)
[90] Cheng et al. 2017	Hong Kong	Newspapers	Real suicides	Werther effect (method specific: charcoal burning)
[91] Ishii 1991	Japan	Newspapers	Real suicides	Werther effect
[92] Stack 1996	Japan	Newspapers	Real suicides	Werther effect (nationality specific)
[93] Ueda et al. 2014	Japan	Newspapers	Real (celebrity) suicides	Werther effect
[94] Shoval et al. 2005	Israel	TV documentary	Real suicide	Mixed results
[95] Bakst et al. 2019	Israel	Newspapers	Real suicides	No effect
[96] Menon et al. 2020	India	Newspapers	Real (celebrity) suicides	Werther effect
[97] Fu and Yip 2009	Hong Kong, Taiwan and South Korea	Newspapers	Real (celebrity) suicides	Werther effect
[98] Holding 1974	United Kingdom	TV series	Fictional suicide	Mixed results
[99] Holding 1975	United Kingdom	TV series	Fictional suicide	Mixed results
[100] Barraclough et al. 1977	United Kingdom	Newspapers	Real suicides	Werther effect
[101] Ashton and Donnan 1979	United Kingdom	Newspapers	Real suicides	Werther effect
[102] Ashton and Donnan 1981	United Kingdom	Newspaper	Real suicides	Werther effect
[103] Ellis and Walsh	United Kingdom	TV soap operas	Fictional suicide	Werther effect
[104] Fowler 1986	United Kingdom	TV soap operas	Fictional suicide	Werther effect
[105] Platt 1987	United Kingdom	TV soap operas	Fictional suicide	No effect
[106] Simkin et al. 1995	United Kingdom	TV dramas	Fictional suicide	No effect
[107] Hawton et al. 1999	United Kingdom	TV series	Fictional suicide	Werther effect
[108] Etzersdorfer et al. 1992	Austria	Newspapers	Real suicides	Papageno effect
[109] Sonneck et al. 1994	Austria	Newspapers	Real suicides	Papageno effect
[110] Etzersdorfer and Sonneck 1998	Austria	Newspapers	Real suicides	Papageno effect
[111] Etzersdorfer et al. 2004	USA	Newspapers	Real suicides	Werther effect
[112] Niederkrotenthaler and Sonneck 2007	Austria	Newspapers	eal suicides	Papageno effect
[113] Jonas 1992	Germany	Newspapers	Real suicides	Werther effect
[114] Kunrath et al. 2011	Germany	Mixed media	Real suicides	Werther effect (method specific: railway suicide)
[115] Ladwig et al. 2012	Germany	Mixed media	Real (celebrity) suicides	Werther effect (method specific: railway suicide)
[116] Saint-Martin et al. 2009	France	Movie	Fictional suicide	Werther effect (method specific: plastic bag asphyxia)
[117] Queinec et al. 2011	France	Mixed media	Real (celebrity) suicides	Mixed results
[118] Hassan 1995	Australia	Newspapers	Real suicides	Werther effect
[119] Hills 1995	Australia	Newspapers	Real (celebrity) suicides	Werther effect
[120] Martin and Koo 1997	Australia	Mixed media	Real (celebrity) suicides	No effect
[121] Pirkis et al. 2006	Australia	Mixed media	Real suicides	Werther effect
[122] Pirkis et al. 2020	Australia	Mixed media	Real (celebrity) suicides	Werther effect (method specific: hanging)
